# Evaluation of High Doses of Phytase in a Low-Phosphorus Diet in Comparison to a Phytate-Free Diet on Performance, Apparent Ileal Digestibility of Nutrients, Bone Mineralization, Intestinal Morphology, and Immune Traits in 21-Day-Old Broiler Chickens

**DOI:** 10.3390/ani12151955

**Published:** 2022-08-02

**Authors:** Beatriz Martínez-Vallespín, Klaus Männer, Peter Ader, Jürgen Zentek

**Affiliations:** 1 Department of Veterinary Medicine, Institute of Animal Nutrition, Freie Universität Berlin, Königin-Luise-Str. 49, 14195 Berlin, Germany; klaus.maenner@fu-berlin.de (K.M.); juergen.zentek@fu-berlin.de (J.Z.); 2 BASF SE, 68623 Lampertheim, Germany; peter.ader@basf.com

**Keywords:** broiler, high phytase doses, performance, intestinal morphology, immune response

## Abstract

**Simple Summary:**

Phytic acid is the main storage form for phosphorus in plants. Although it is the main source of organic phosphorus in broiler diets, its utilization by monogastric animals is limited. In addition, phytic acid displays antinutritional effects, impacting optimal nutrient and energy digestibility. The supplementation of feed with phytases enables broilers to use phytate phosphorus more efficiently. Superdosing of phytase has been reported to have additional beneficial effects on the animal beyond the ones derived from the improved phosphorus utilization. This study tried to elucidate the mechanisms related to the immune response and mucosal morphology contributing to those overall beneficial effects. Although addressing performance was not the primary target of the study, performance improved linearly with increasing levels of phytase. The use of increasing doses of phytase showed linear increases in apparent ileal digestibility of crude protein, crude ash, and phosphorus. Jejunal crypt depth decreased with the addition of phytase to a low-protein diet, as well as the content of CD3-positive intraepithelial lymphocytes, but the results could not demonstrate clear differences between phytase doses in these specific parameters.

**Abstract:**

The supplementation of feed with phytases enables broilers to utilize more efficiently phosphorus (P) from phytic acid (IP_6_), the main storage form of P in plants. The current study evaluated the addition of 500, 1000, and 3000 FTU of phytase per kg to a phytate-containing diet with low P level (LP) fed to broilers from 1 to 21 days of age and compared it to a hypoallergenic phytate-free diet (HPF). There was a linear improvement in performance parameters with increasing levels of phytase in the LP diet (*p* < 0.001). Apparent ileal digestibility of crude protein, P, and some amino acids, increased with phytase. Crude ash, P, and the calcium content of tibia bones linearly increased with increasing levels of phytase (*p* < 0.001). Crypt depth (related to body weight) in the jejunum epithelium linearly decreased with phytase addition (*p* < 0.001). Cecal crypt depth decreased with phytase supplementation (*p* = 0.002). Cecum tissue showed lower counts of CD3-positive intraepithelial lymphocytes in broilers receiving the phytase in comparison to LP (*p* < 0.001), achieving similar counts to HPF-fed broilers. Although results from the current study seem to point out some mechanisms related to the immune response and mucosal morphology contributing to those overall beneficial effects, no clear differences between different phytase doses could be demonstrated in these specific parameters.

## 1. Introduction

Phytic acid (myo-inositol 1,2,3,4,5,6-hexakis dihydrogen phosphate; IP_6_) and its salts (phytates) are the main sources of organic phosphorus (P) in broiler diets, but the utilization of that P is rather limited. In order to make the P from IP_6_ available for monogastric animals, phosphate groups need to be cleaved from the inositol ring [1]. The supplementation of feed with dietary, microbially produced extrinsic phytases (Phy) enables broilers to hydrolyze the IP_6_ to lower inositol phosphate esters (IP_5_ to IP_1_) and utilize inorganic P more efficiently, thus improving the digestibility of P [2]. Another important application of this technology is the possibility of reducing the addition of inorganic sources of non-phytate P to the diets which would lead to lower P excretion, diminishing its negative impact on the environment [3].

The inability of broilers to use the phytate-P efficiently is not mainly related to a lack of Phy activity along the gastrointestinal tract but to the low rate of hydrolysis of the higher IP esters in the small intestine, usually impaired by supplementing the diets with calcium (Ca) and P [4,5]. IP_6_ disappearance in the hindgut of broilers fed a Phy unsupplemented diet has been found to be up to 91%, although the P absorption posterior to the ileum is negligible [6,7]. Thus, degradation of IP_6_ in an animal’s stomach is desirable through the dietary inclusion of Phy. It should be noted that Phy is not specific for IP_6_ and, therefore, further hydrolysis to lower IP esters can be expected [1].

Not only does IP_6_ limit the availability of the P present in plant-based ingredients, but it also exhibits some anti-nutritional effects. In fact, IP_6_ shows chelating potential, being able to bind to different minerals or proteins, forming insoluble salts or phytate-protein complexes, respectively [8,9,10]. Moreover, an increase in endogenous amino acid (AA) losses due to the increase in digestive enzyme and mucin secretion was described [10,11]. Thus, the use of dietary Phy might directly or indirectly affect morphological parameters and physiological processes of the intestinal mucosa.

The doses of Phy considered conventional and commonly used in broiler production are in the range of 500 to 1000 FTU/kg feed. A considerable number of studies on monogastric nutrition testing higher Phy inclusions (often called “superdoses”) have observed that there seem to be dose-dependent effects on feed efficiency and performance [12,13,14,15,16] and, furthermore, these seem to be beyond the effects related to the higher release of phytate-P only [4]. However, the mechanisms involved in the reported positive effects of superdoses of Phy are not completely understood and need further investigation. Thus, the purpose of this study was to evaluate the effects of increasing levels of dietary Phy on performance, apparent ileal digestibility (AID), bone mineralization, and predominantly, on gut function and mucosal responses in 21-day-old broilers.

## 2. Materials and Methods

### 2.1. Animals and Experimental Design

Procedures involving animal handling and treatment were approved by the State Office of Health and Social Affairs Berlin (LAGeSo, Berlin, Germany Reg. No. 0439/17).

A total of 140 one-day-old male broiler chickens (Cobb 500) were obtained from a local hatchery (Cobb Germany Avimex GmbH, Wiedemar, Germany). The broiler chickens were sexed and vaccinated against infectious bronchitis and Newcastle disease at the hatchery. After arrival, chickens were randomly allocated to 70 metabolic cages in a climate-controlled poultry house using two birds per cage (14 replicates per treatment). The cages measured on average 0.38 × 0.52 m, providing 0.10 m^2^ per animal.

The poultry house was provided with artificial light (30 lux), a controlled climate, and forced ventilation (0.4–0.5 m^3^/h/kg body weight). The barn was pre-warmed for 48 h prior to poultry placement. The temperature was kept at 32 °C during the first week of the trial and was gradually diminished to 29 °C and 26 °C during the second and third weeks, respectively. The artificial light was kept on for 24 h during the four first days and from day 5 the lighting regime consisted of an 18 h light and 6 h dark cycle. Birds had ad libitum access to feed in mash form and water throughout the whole experiment.

Two basal diets were formulated for feeding during the 21-d period. The first diet was a “hypoallergenic” phytate-free diet (HPF) based on insect meal from *Hermetia illucens* as the protein source and corn starch as the carbohydrate. The second diet was a phytate-containing diet with a low P content (LP) that was formulated based on corn, soybean meal, and sunflower meal. The LP diet was supplemented with three different concentrations of microbial Phy (Natuphos^®^ E, BASF SE, Ludwigshafen, Germany): 500, 1000, and 3000 FTU/kg feed. The diets were formulated to meet or slightly exceed the nutrient requirements for broiler chickens recommended by the Society of Nutritional Physiology (GfE, 1999) with the exception of metabolizable energy (ME), crude protein (CP), AA, and P. The matrix values for the dose rate of 1000 FTU/kg feed (supplied by BASF SE) of the added enzyme for these nutrients and ME were considered and LP was formulated for ME of 0.65 MJ/kg, for CP and AA of approximately 3%, and for P at 2 g/kg below the requirements recommended. The Ca content was in accordance with the recommended minimum specifications given by the breeder (9 g/kg). Natuphos^®^ E was included in the phytate-containing diet at the expense of Tixosil (silicon dioxide >97%; Solvay GmbH, Hannover, Germany). In addition, Titanium (IV)-dioxide (TiO_2_) was added as a marker for AID measurements at a dose level of 3 g/kg. The composition of the diets is shown in Table 1.

All broiler chickens were observed twice per day for any abnormalities, abnormal behavior, or clinical signs of sickness throughout the experimental period and morbidity and mortality were recorded. At the end of the experiment, all birds were stunned and killed by exsanguination for sampling.

### 2.2. Performance Parameters

Body weight (BW) and feed consumed by the birds were recorded weekly. Thus, body weight gain (BWG), feed intake (FI), and feed conversion ratio (FCR) for days 7, 14, and 21 were calculated. In addition, the European Poultry Efficiency Factor (EPEF) was calculated. EPEF is a value that standardizes technical results, considering FCR, mortality/culling, and BW, and it was calculated as follows:

$$\mathrm{EPEF}=\frac{\mathrm{Average}\;\mathrm{daily}\;\mathrm{BWG}\;\left(g\right)\times \%\;\mathrm{survival}\;\mathrm{rate}}{\mathrm{FCR}\;\times 10}$$

### 2.3. Collection of Samples

Ileal digesta from the posterior half between Meckel’s diverticulum and 2 cm before the ileo-ceco-colonic junction was collected and stored at −20 °C before being freeze-dried for chemical analysis. Data from the two birds in a cage were pooled resulting in 14 replicates per treatment. The AID of nutrients was calculated using the following formula:

$$\mathrm{AID}\;\left(\%\right)=100-\left(\frac{\%\;\mathrm{marker}\;\mathrm{in}\;\mathrm{feed}}{\%\;\mathrm{marker}\;\mathrm{in}\;\mathrm{ileum}}\times \frac{\%\;\mathrm{nutrient}\;\mathrm{in}\;\mathrm{ileum}}{\%\;\mathrm{nutrient}\;\mathrm{in}\;\mathrm{feed}}\right)\times 100$$

With such small animals, especially in the case of the HPF group, the amount of digesta collected was very little and it had to be further pooled resulting, therefore, in only three and six replicates in the HPF and LP groups, respectively. This certainly can be considered a limitation, but on the other hand, we think that some interesting observations should still be shared.

The mid jejunum and cecum were taken immediately and preserved by formalin fixation for morphological measurements and intraepithelial lymphocytes (IELs) count performed by histology. The left tibia bone was excised, and the soft tissue was removed. After weighing (as is), tibia bones were freeze-dried and pooled (2 birds each) for subsequent chemical analyses.

### 2.4. Histological Procedures

The jejunum and cecum sections were fixed in 4% phosphate-buffered formaldehyde (Carl Roth GmbH, Karlsruhe, Germany), dehydrated in increasing concentrations of ethanol (from 70% to 96%) and isopropanol, and cleaned with xylol. The samples were then immediately embedded in paraffin wax, and five µm sections were cut with a sledge microtome (Type 1400, Leitz, Wetzlar, Germany), mounted on glass slides, and dried in an incubator at 37 °C. Before staining, the sections were deparaffinized with xylol and rehydrated with decreasing concentrations of ethanol. Hematoxylin/eosin staining was performed allowing the measurement of the villus length (jejunum) and crypt depth (jejunum and cecum) using a light microscope (Olympus BX43, Olympus Co., Tokyo, Japan) equipped with a digital camera (DP72, Olympus, Hamburg, Germany). The measurements were taken using cellSens imaging software (v. 1.4, Olympus, Hamburg, Germany).

Immunohistochemical analyses were also performed on the cecum sections to determine the CD3-positive intraepithelial lymphocytes (IELs). Once the sections were deparaffinized and rehydrated, they were pre-treated with a 3% hydrogen peroxide solution to block endogenous peroxidase. Antigen retrieval was performed by heating sample sections in citrate buffer (pH 6) at 121 °C for 5 min in an autoclave. To avoid the non-specific binding of antibodies, a protein block was used for 10 min, prior to incubation with a primary antibody. Tissue sections were then incubated with rat anti-human CD3 (1:100; AbD Serotec, Düsseldorf, Germany) as a primary antibody at 4 °C overnight in humidity chambers. After washing with PBS buffer, tissue sections were incubated with mouse anti-rat IgG1 conjugated with horseradish peroxidase (1:500; Southern Biotech, Birmingham, AL, USA) as a secondary antibody for 45 min at room temperature. To visualize the primary antibody, sections were stained with DAB chromogen (3,3′ diaminobenzidine). Further staining with hematoxylin was also performed. The number of CD3-positive IELs was counted with the light microscope in 10 well-oriented crypts per animal and the results were expressed per 10,000 µm^2^.

### 2.5. Chemical Analyses

Chemical analyses of feed samples included Weende constituents and, additionally, AA, starch, total sugars, Ca, P, and sodium. Analyses were in accordance with the methods issued by VDLUFA (dry matter: VDLUFA III 3.1; CP: VDLUFA III 4.1.1 modified according to macro-N determination (vario MAX CN); AA: VDLUFA III 4.11.1; crude fiber: VDLUFA III 6.1.4; crude ash: VDLUFA III 8.1; crude fat: VDLUFA III 5.1.1; starch: VDLUFA III 7.2.1; total sugars: VDLUFA III 7.1.1; Ca, P and sodium: VDLUFA VII 2.2.2.6). Samples were also analyzed to confirm the concentrations of Phy activity (ISO 30024:2009) and phytate P content [17]. Pooled ileal samples were ground to pass through a 0.25 mm screen and analyzed for CP, crude ash, Ca, P, and AA. Tibia bones were ground to pass through a 0.25 mm screen and analyzed for ash, P, and Ca in accordance with previously cited methods given by VDLUFA. For minimizing the effect of individual fat concentration on mineral level, bones were defatted (VDLUFA III 5.1.1) before chemical analyses. TiO_2_ was measured in feed and ileal digesta according to the method described by Short et al. [18].

### 2.6. Statistical Analyses

Data were analyzed using one-way analysis of variance (ANOVA) with the diet as the fixed factor, followed by posthoc Tukey´s test using the IBM SPSS Statistics for Windows, version 27 (IBM Corp., Armonk, NY, USA). Non-normally distributed data were analyzed using the Kruskal–Wallis H test. The cage represented the experimental unit. Linear and quadratic regression analyses were also performed to test the effect of the increasing levels of Phy. Results are reported as mean values followed by SEM. Differences were considered significant when *p* ≤ 0.05.

## 3. Results

### 3.1. Performance Parameters

Broiler chickens were healthy during the study. Three animals distributed in different groups died of anorexia during the four first days of the trial and two others had to be culled due to mobility problems at 9 and 13 days of age. Thus, the overall mortality rate with the inclusion of culling amounted to 3.56% and treatment effects could be excluded.

Initial BW was 44.1 ± 0.2 g without differences among treatment groups (*p* = 1.000; Table 2). Animals in the HPF group showed very low feed intake and growth traits during the whole experiment therefore no comparisons with the LP groups will be done in this section. BWG showed important differences resulting in higher final BW in all the animals receiving the Phy, in comparison with the LP group (*p* < 0.001). Birds receiving the highest Phy dose registered the highest FI and had the highest BW at the end of the trial (*p* = 0.001). Moreover, FCR decreased with the addition of the Phy (*p* < 0.001). Finally, EPEF clearly improved with the addition of Natuphos^®^ E (+34% with 500 and 1000 FTU/kg and +59% with 3000 FTU/kg Phy; *p* < 0.001).

### 3.2. Apparent Ileal Digestibility

Results of the AID of gross energy (GE), some selected nutrients, and AA are shown in Table 3. Animals in the HPF groups showed the highest AID of GE and phosphorus (*p* < 0.001) while the AID of CP was lower than that registered by the groups receiving the Phy, (*p* < 0.001). The AID of GE and CP was significantly higher due to Phy only in the LP + 1000 group. On the other hand, the animals receiving 1000 or 3000 FTU/kg of Phy showed higher AID of crude ash and P than the LP group. Dose-dependent effects on AID (with the exception of Ca) were observed through significant effects of linear (CP, crude ash, P) and quadratic (GE, CP, P) regressions (*p* < 0.01).

The HPF group showed similar AID of AAs to the LP group, with the exception of Glu which was lower (*p* < 0.001). The inclusion of 1000 or 3000 FTU/kg of Natuphos^®^ E in the LP diet did generate improvements in the AID of Ala, Cys, Glu, Gly, His, Leu, Met, and Ser (*p* < 0.05), while the lowest dose (500 FTU/kg feed) only improved the AID of Met in comparison to the LP group (+12.9%; *p* < 0.001).

### 3.3. Tibia Mineralization

Animals in the HPF group showed lower tibia bone weight than the animals receiving the Phy, especially in comparison with the animals receiving the highest dose (−14.6% “as is”, and −47.2% “dried”, respectively, *p* < 0.001; Table 4). Increasing Natuphos^®^ E inclusion rates improved tibia bone weights significantly, by up to 32.6% (as is) and 33.3% (dried) in the LP + 3000 group compared to the LP.

Crude ash, P, and Ca content increased linearly with supplementation of Phy compared to LP (*p* < 0.001). Crude ash content in the HPF group was similar to the groups receiving the Phy (*p* < 0.001). P content in HPF was higher than in LP while Ca was only significantly different and lower than LP + 3000 (*p* < 0.001). Except for P, the other parameters were not improved by the addition of 1000 or 3000 FTU/kg feed compared to the treatment supplemented with 500 FTU/kg feed. In the case of P, there was a dose-related difference, with animals receiving 1000 or 3000 FTU/kg showing higher tibia P concentrations than the ones receiving 500 FTU/kg (*p* < 0.001).

### 3.4. Histological Findings in the Intestinal Tissue

Morphological measurements of the jejunum and cecum were expressed in relation to BW, due to the different BW registered at the end of the trial (Table 5).

The HPF group had the highest villus length and crypt depth per BW in jejunal tissue (*p* < 0.001). The addition of Phy to LP reduced the crypt depth per BW (*p* < 0.001) whereas reduction at dose rates 1000 and 3000 FTU/kg feed was only numerical compared to treatment LP + 500. Regarding the villus/crypt ratio (V:C), the HPF group showed the numerically highest value, although it was not significantly different from the values registered with the two highest doses of Phy. Increasing levels of Phy showed a linear increase in V:C. In the cecum, the HPF and LP groups showed the highest values for crypt depths. Graded levels of Phy decreased crypt depth with the highest dose rate (3000 FTU/kg feed) showing a significant reduction of crypt depth of 31.7% compared to LP (*p* < 0.005).

**Figure 1:**
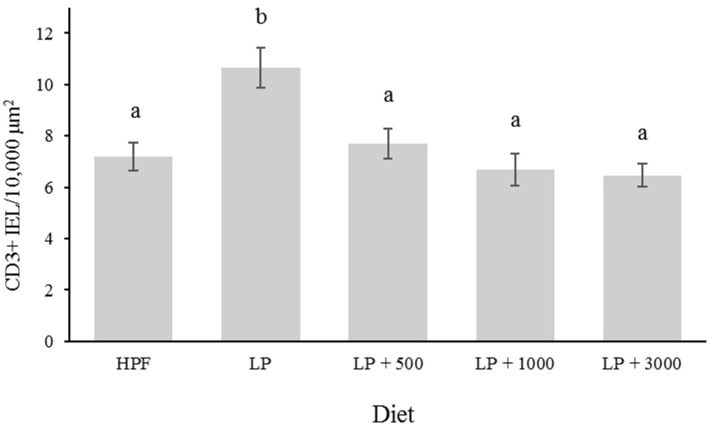
Effect of different inclusion levels of phytase on CD3-positive intraepithelial lymphocytes in cecum of broiler chickens at 21 days of age (*p* < 0.001); HPF: hypoallergenic phytate-free diet; LP: low-phosphorus diet; LP + 500: low-phosphorus diet + 500 FTU/kg feed; LP + 1000: low-phosphorus diet + 1000 FTU/kg feed; LP + 3000: low-phosphorus diet + 3000 FTU/kg feed. ^a,b^ Bars not sharing any common superscript are significantly different.

In regard to the CD3-positive IELs count in the cecum (Figure 1), animals in LP showed the highest cell density compared to all other treatments (*p* < 0.001). The supplementation of increasing doses of Natuphos^®^ E induced a linear decrease of CD3-positive IELs (*p* < 0.001) that achieved the same level as was recorded in HPF.

## 4. Discussion

The current study shows that the use of dietary Phy in broiler feed improved performance and nutrient digestibility, which is consistent with previous observations [16,19,20]. Although addressing performance was not the primary target of the study, it is interesting to note that performance improved linearly with increasing levels of Phy.

The HPF diet showed a negative effect on the animals, related probably to its organoleptic characteristics, which led to a lower FI and reduced growth during the whole trial. Thus, due to the big differences in growth performance, results from the HPF group regarding these parameters will no longer be discussed.

It should be also mentioned that performance results obtained in the current trial are beneath those expected according to the objectives from the breeder. One reason for this may have been the fact that the diets were low in P. On the other hand, as commented above, the main focus of the trial was the study of the effect of the Phy on intestinal parameters, so the number of animals used in each replicate (i.e., two birds), as well as the use of metabolic cages, following the reduction principle from Animal Welfare recommendations, would have also led to some unusual performance data. Usually, the addition of Phy implicates a nutrient and energy reduction in the diet formulation based on the expected improved nutrient and energy utilization triggered by the enzyme at different dose rates. The addition of 500 FTU/kg has shown beneficial effects mostly in nutrient-reduced diets (negative control) [16,20,21,22]. Sommerfeld et al. [23] observed a slightly higher average daily gain in broilers up to 11 days of age when adding 500 FTU/kg to a diet low in P and Ca, in comparison with a positive control diet fulfilling the nutrient requirements, but the effect was diluted during the whole experimental period of 22 days.

A recent study using similar doses to the ones in the current study showed an increase in BW when using 1500 and 3000 FTU/kg in comparison with 500 FTU/kg, in low P and Ca diets, but no differences were observed between the two highest inclusions [24]. Many other studies support the results from the current study and have shown a linear effect of the Phy dose in some performance parameters such as BWG or FI [16,25]. One of these studies showed an increase in FI with doses between 750 and 3000 FTU/kg used in a low P diet, even in comparison with a positive control. Moreover, an increase in BW was also observed with 2000 and 3000 FTU/kg feed [16]. Nevertheless, the results obtained with the inclusion of Phy in nutrient-reduced diets are usually comparable to those of the corresponding positive controls [13,20,26].

The addition of Phy linearly improved nutrient digestibility in comparison with the negative control, with the exception of Ca which was similar in all groups. The main objective of the use of dietary Phy in broiler feed is the increase of P utilization and this effect has been consistently observed by different studies, but the impact on Ca is more variable. Higher ileal Ca digestibility has been observed in low P diets already with 500 FTU/kg Phy [27,28]. The same effect was reported in diets independent of the level of phytic acid (low, medium, or high) with values between 500 and 1000 FTU/kg Phy [29]. On the contrary, a study using 500 and 1500 FTU/kg Phy in both a positive and a negative control showed decreases in ileal Ca digestibility in enzyme supplemented diets, with the exception of the 500 FTU/kg dose in the negative control which had similar values to the diet without Phy [21]. The authors attributed this effect to the increase in Ca release following phytate hydrolysis, widening the Ca:available P (aP) ratio, and the subsequent saturation of absorption, which could also affect other nutrients. Other factors could be also involved, such as the quality of the limestone that could lead to complexations with other nutrients, impacting the Ca digestibility [30].

Regarding the AID of AAs, again, the HPF group showed results that are difficult to compare with the other groups, showing for some AAs a lower level of AID than for the LP group. The reasons for these unexpected effects, apart from the already mentioned low replication number, may be lower bio availabilities of amino acids from *Hermetia* meal and/or higher endogenous secretion due to its chitin content [31].

The AID of CP and some AAs was improved with Phy supplementation. An important part of the dietary protein is susceptible to forming complexes with phytate, although the extent of the phytase response may be dependent on many factors, such as the diet composition, the solubility of phytate, or the Ca concentration [32,33]. A complexation of phytate with intrinsic digestive enzymes is also hypothesized [34].

The positive effect of the Phy on AA digestibility is generally associated with the decrease of endogenous losses [35]. This decrease could be due to the direct effect of the Phy itself or to the increase in FI [36], as a relationship has been established between FI and endogenous AA losses [37].

The current results show that the most pronounced improvement in the AID of AAs was observed at a dose of 1000 FTU/kg, while values were rather similar to those found at 3000 FTU/kg. According to a meta-study on the effect of Phy on ileal AA digestibility in broilers, higher improvements are observed with inclusions between 500 and 1000 FTU/kg, while at higher dose rates a plateau was postulated by the authors [35]. However, when comparing studies, there is not always such a clear effect on AID. Thus, very limited improvement in AA digestibility was observed in some studies using standard doses but also some so-called superdoses of Phy (2500 and 5000 FTU/kg feed) in broilers [28,38]. In fact, the comparison of studies is sometimes difficult because they differ in important aspects such as the composition of the diets P inclusion, Ca/P-ratio, and Phy used.

Results regarding the AID of AAs from the current study are lower in comparison with other studies [35]. Although no clear explanation for that can be offered, it can be speculated that the importance of endogenous secretion at lower feed intakes and appropriately formulated diets with, for example, higher levels of sunflower extract meal, contributed to the results. Thus, the AID of some AAs such as Gly, Met, Ser, and Thr shows very low values only in the LP group, and in the case of Gly, Ser, and Thr also in the LP + 500 group. Ravindran et al. [39] measured the AID of AAs in sunflower meal in 42-day-old broilers, using this feedstuff as the only protein source. The AID of Gly, Thr, and Ser was much lower than the one registered in the same study using soybean meal as the only protein source (−21%, −15%, and −6.8%, respectively). In the current study, the inclusion of sunflower meal may have been high enough (12%) to observe a lower AID of the cited AAs, but the inclusion of high levels of Phy (1000 and 3000 FTU/kg feed) may have been able to counteract that effect.

Most of the Ca and P body content is present in the skeleton [40] and, therefore, the analysis of bone mineralization parameters can be used to study the Ca and P absorption. The results from the current study show that the use of Phy increased tibia ashes as well as P and Ca tibia content, even when the AID of Ca was not affected by Phy addition. These observations are in agreement with previous findings in low P diets similar to the ones used in the current study [19,20,41]. On the other hand, it is remarkable that the AID of some AAs was still increased at dose levels beyond 500 FTU/kg feed, while bone parameters (except P percentage in bone ash) were not further improved by higher Phy levels. This might indicate an effect that is not dependent on the animal´s requirement for P.

The limited number of studies and the differences in experimental design and diet composition, or the studied intestinal portions, make it difficult to obtain clear conclusions on the possible effects of the Phy addition on gut morphology.

In general, villus length is related to the absorption capacity in that the longer the villi, the higher the absorption. On the other side, the crypt depth is related to cell turnover and deeper crypts would indicate a rise in cell renewal due to epithelial destruction. Thus, a higher V:C would be an indicator of a more efficient absorptive function [42].

A study using diets meeting the P requirements observed that the use of 500 FTU/kg led to an increase in duodenum villus length and a decrease in jejunum crypt depth [43]. Another study found that the inclusion of 500 FTU/kg in low P diets increased jejunum villus length which achieved a similar level to the positive control [44]. The inclusion of 500 FTU/kg Phy in low P diets was also reported by Emami et al. [45] to increase V:C in all parts of the small intestine. Higher levels of Phy have also been tested. Thus, the use of 1000 FTU/kg Phy led to an increase in jejunum villus length in a low P diet, while the effect was the opposite when Phy was added to diets with an adequate P level [46].

The results from the current study show that, even if the animals receiving the phytate-free diet did not grow as expected due to the lower FI, this diet was more beneficial to the jejunal and cecal epithelium than LP. Supplementation of LP with 1000 and 3000 FTU Phy/kg feed seemed to improve the epithelial morphology when comparing V:C, reaching values closer to those of the HPF group. The reduction of crypt depth in the jejunum and cecum by graded levels of Phy also supports that hypothesis. These findings are rather likely related to the degradation of phytate and its metabolites by the increasing levels of Phy. On the other hand, animals receiving the HPF diet registered the highest crypt depth. It could be hypothesized that due to the low feed intake registered in this group, an increase in cell proliferation would be a response to increasing mucosal surface in the hindgut as an attempt to compensate for the low nutrient uptake.

The gut-associated lymphoid tissue is the first line of immune defense in mucosal tissue and the mobilization of T-cells and their infiltration into the tissue is one of its strategies. The IELs expressing the CD3 polypeptide complex are the most abundant in adult chickens, although their percentage depends on age with reported values of CD3-positive cells of around 63% of total intestinal lymphocytes in 4-week-old chickens [47]. To the author´s knowledge, there is no information available on the effect of phytate-containing diets and their supplementation with Phy on immune response in broiler´s cecal mucosa. The results obtained in the current study indicate that there was an increased immune response in the animals in the LP group that was reduced by the inclusion of Phy at any dose, reaching values similar to the ones registered in the HPF group. The presence of a greater amount of the higher IP esters in the intestinal digesta might directly or indirectly unleash an immune response in the LP group. Lower IP esters (≤IP_4_) could still possess important antinutritional properties [48]. Thus, the use of superdoses of Phy would allow the hydrolyzation of IP_6_ to rather low IP esters and thereby potentially reduce the pro-inflammatory response [24].

## 5. Conclusions

The feeding of broilers from day 1 to 21 of their lives with feed supplemented with 1000 or 3000 FTU Phy/kg decreased the antinutritional effects of IP_6_ and its lower esters which resulted in better performance results, nutrient digestibility, and bone mineralization. Although the results from the current study seem to point out some mechanisms related to the immune response and mucosal morphology contributing to those overall beneficial effects, no clear differences between different Phy doses could be demonstrated for these specific parameters. Further studies need to be conducted to clarify the effects of the degradation of IP_6_ and its lower esters by supplementing phytate-containing diets with high Phy doses. A special focus should be on the study of the potential effects on physiological and immunological processes and their impact on broiler performance.

## Figures and Tables

**Table 1:** Ingredients and nutritional characteristics of the diets (as is).

Treatment ^1^		HPF	LP	LP + 500	LP + 1000	LP + 3000
		Ingredients (%)				
Corn			54.04	54.04	54.04	54.04
Corn starch		57.34				
Soybean meal			26.13	26.13	26.13	26.13
*Hermetia illucens* meal		37.60				
Sunflower meal			12.00	12.00	12.00	12.00
Soybean oil		0.80	3.87	3.87	3.87	3.87
Limestone		1.51	1.93	1.93	1.93	1.93
Monocalcium-phosphate		1.10	0.21	0.21	0.21	0.21
Premix ^2^		1.20	1.20	1.20	1.20	1.20
L-Lysine–HCL			0.13	0.13	0.13	0.13
DL-Methionine		0.15	0.09	0.09	0.09	0.09
L-Tryptophan			0.04	0.04	0.04	0.04
Titanium dioxide		0.30	0.30	0.30	0.30	0.30
Tixosil ^3^			0.06	0.05	0.04	
Natuphos^®^ E 5000 G				0.01	0.02	0.06
		Calculated composition				
ME ^4^	MJ/kg	12.35	12.35	12.35	12.35	12.35
Crude protein	%	22.27	22.27	22.27	22.27	22.27
Crude fiber	%	4.51	4.48	4.48	4.48	4.48
Crude ash	%	5.65	5.21	5.21	5.21	5.21
Ether extract	%	2.83	6.74	6.74	6.74	6.74
Lysine	%	1.81	1.20	1.20	1.20	1.20
Methionine	%	0.46	0.46	0.46	0.46	0.46
Tryptophan	%	0.29	0.29	0.29	0.29	0.29
Threonine	%	0.80	0.84	0.84	0.84	0.84
Calcium	%	0.90	0.90	0.90	0.90	0.90
Phosphorus	%	0.70	0.50	0.50	0.50	0.50
Phytase activity	FTU/kg	-	-	500	1000	3000
		Analyzed composition				
Dry matter	g/kg	901.2	897.5	900.3	898.1	897.6
Crude protein	g/kg	231.2	223.5	224.2	225.6	224.4
Crude fiber	g/kg	40.6	46.5	47.3	48.4	46.8
Crude ash	g/kg	55.8	52.7	51.9	52.0	51.8
Ether extract	g/kg	30.5	66.5	67.1	65.9	66.9
Starch	g/kg	534.0	382.2	380.4	376.4	378.2
Total sugars	g/kg	49.5	51.2	51.0	50.9	50.2
Calcium	g/kg	9.2	8.8	8.9	8.6	8.7
Phosphorus	g/kg	7.5	4.5	4.4	4.6	4.5
Sodium	g/kg	1.8	1.9	2.0	1.9	1.9
Phytase activity	FTU/kg	<60	<60	575	1227	3502
Phytate phosphorus	g/kg	<0.1	2.82	2.77	2.73	2.73

^1^ LP: phytate-containing diets low in phosphorus; ^2^ Contents per kg: 600,000 I.U. Vit. A (acetate); 120,000 I.U. Vit. D_3_; 6000 mg Vit. E (α-tocopherol acetate); 200 mg Vit. K_3_ (MSB); 250 mg Vit. B_1_ (mononitrate); 420 mg Vit. B_2_ (cryst. riboflavin); 300 mg Vit. B_6_ (pyridoxin-HCl); 1500 μg Vit. B_12_; 3000 mg niacin (niacinamide); 12,500 μg biotin (commercial, feed grade); 100 mg folic acid (cryst., commercial, feed grade); 1000 mg pantothenic acid (Ca d-pantothenate); 60,000 mg choline (chloride); 5000 mg iron (iron carbonate); 5000 mg zinc (zinc sulfate); 6000 mg manganese (manganous oxide); 1000 mg copper (copper oxide); 45 mg iodine (calcium-iodate); 20 mg selenium (sodium-selenite); 140 g sodium (NaCl); 55 g magnesium (magnesium sulfate); carrier: calcium carbonate (calcium min 38%); ^3^ Silicon dioxide >97%; ^4^ Estimated according to equation of WPSA 1984 (by using crude nutrients).

**Table 2:** Effect of different inclusion levels of phytase on growth performance of broiler chickens.

							*p*-Value		
Treatment Groups ^1^	HPF	LP	LP + 500	LP + 1000	LP + 3000	SEM		Linear ^2^	Quadratic ^3^
Replicates ^4^	14	14	14	14	14				
Initial body weight (g)	44.1	44.1	44.0	44.1	44.0	0.23	1.000		
Final body weight (g)	418 ^d^	600 ^c^	679 ^b^	693 ^b^	772 ^a^	15.7	<0.001	<0.001	<0.001
Body weight gain (g)	374 ^d^	556 ^c^	635 ^b^	649 ^b^	728 ^a^	11.02	<0.001	<0.001	<0.001
Feed intake (g)	500 ^c^	848 ^b^	906 ^ab^	920 ^ab^	978 ^a^	12.05	<0.001	<0.001	<0.001
Feed conversion ratio ^5^	1.338 ^c^	1.530 ^a^	1.428 ^a^	1.418 ^ab^	1.348 ^bc^	0.016	<0.001	<0.001	<0.001
EPEF ^6^	134 ^b^	158 ^b^	213 ^a^	211 ^a^	252 ^a^	7.29	<0.001	<0.001	0.525

^1^ HPF: hypoallergenic phytate-free diet; LP: phytate-containing diets low in phosphorus; ^2^ Linear effects of supplementing increasing levels of phytase; ^3^ Quadratic effects of supplementing increasing levels of phytase; ^4^ Data are means of 14 cages with 2 birds per cage; ^5^ Kruskal–Wallis test; ^6^ European poultry efficiency factor: averaged grams gained per day × survival rate (%) ÷ feed conversion ratio × 10; ^a,b,c,d^ Within a row, means not sharing any common superscript are significantly different (*p* < 0.05).

**Table 3:** Effect of different inclusion levels of phytase on apparent ileal digestibility of main nutrients and amino acids in broiler chickens at 21 days of age.

							*p*-Value		
Treatment Groups ^1^	HPF	LP	LP + 500	LP + 1000	LP + 3000	SEM		Linear ^2^	Quadratic ^3^
Replicates ^4^	3	6	12	12	12				
		Nutrients (%)							
Gross energy	78.27 ^a^	64.27 ^c^	66.52 ^bc^	71.29 ^b^	68.86 ^bc^	0.72	<0.001	0.925	0.006
Crude protein	66.25 ^c^	72.12 ^bc^	76.74 ^ab^	80.43 ^a^	78.67 ^ab^	0.76	<0.001	<0.001	0.004
Crude ash	41.24 ^ab^	38.13 ^b^	43.12 ^ab^	44.21 ^a^	46.75 ^a^	0.65	<0.001	<0.001	0.438
Calcium	59.48	55.04	56.74	58.13	58.45	0.55	0.219	0.242	0.231
Phosphorus	65.48 ^a^	37.46 ^c^	41.11 ^c^	54.58 ^b^	60.12 ^b^	1.56	<0.001	<0.001	<0.001
		Amino acids (%)							
Alanine	77.16 ^ab^	73.12 ^b^	78.35 ^ab^	81.83 ^a^	79.58 ^ab^	0.93	0.017	0.017	0.810
Arginine	83.88 ^b^	85.94 ^ab^	87.62 ^ab^	89.85 ^a^	89.01 ^a^	0.60	0.013	0.003	0.221
Aspartic acid	80.98	79.64	77.53	83.46	83.50	0.88	0.160	0.066	0.180
Cysteine	53.75 ^b^	47.76 ^b^	56.60 ^b^	68.06 ^a^	71.38 ^a^	1.98	<0.001	<0.001	0.090
Glutamic acid	78.43 ^c^	83.30 ^b^	85.49 ^ab^	88.79 ^a^	87.82 ^ab^	0.74	<0.001	<0.001	0.019
Glycine	60.86 ^b^	63.58 ^b^	68.08 ^ab^	74.87 ^a^	74.61 ^a^	1.38	0.001	<0.001	0.546
Histidine	72.68 ^b^	72.45 ^b^	76.55 ^ab^	84.09 ^a^	82.99 ^a^	1.20	<0.001	<0.001	0.883
Isoleucine	81.90	74.32	77.75	79.43	79.69	0.97	0.198	0.407	0.205
Leucine	76.64 ^ab^	74.89 ^b^	77.18 ^ab^	82.62 ^a^	81.28 ^a^	0.82	0.003	0.001	0.778
Lysine	76.51 ^b^	80.39 ^ab^	82.41 ^ab^	86.22 ^a^	82.88 ^ab^	0.81	0.011	0.006	0.032
Methionine	85.64 ^a^	76.79 ^b^	86.69 ^a^	88.30 ^a^	87.45 ^a^	1.00	<0.001	0.001	0.764
Phenylalanine	80.51 ^c^	80.55 ^bc^	81.62 ^abc^	85.81 ^ab^	86.01 ^a^	0.69	0.001	<0.001	0.612
Proline	75.44	78.91	76.35	80.08	79.86	0.86	0.477	0.174	0.975
Serine	75.6 ^ab^	72.32 ^b^	72.53 ^b^	80.47 ^a^	80.01 ^a^	0.98	0.001	0.001	0.108
Threonine	69.12 ^ab^	64.56 ^b^	67.09 ^ab^	75.20 ^a^	73.57 ^ab^	1.18	0.004	0.003	0.366
Tyrosine	81.19	76.37	75.27	81.25	82.49	0.92	0.130	0.034	0.019
Valine	61.23 ^b^	68.91 ^ab^	76.43 ^a^	72.97 ^a^	71.64 ^a^	1.26	0.009	0.038	0.005

^1^ HPF: hypoallergenic phytate-free diet; LP: phytate-containing diets low in phosphorus; ^2^ Linear effects of supplementing increasing levels of phytase; ^3^ Quadratic effects of supplementing increasing levels of phytase; ^4^ Data are means of 14 cages with 2 birds per cage (pool of the 2 animals; due to low amounts of digesta samples in HPF and LP groups, ileal digesta was further pooled resulting in only 3 and 6 replicates, respectively); ^a,b,c^ Within a row, means not sharing any common superscript are significantly different (*p* < 0.05).

**Table 4:** Effect of different inclusion levels of phytase on tibia mineralization of broiler chickens at 21 days of age.

							*p*-Value		
Treatment Groups ^1^	HPF	LP	LP + 500	LP + 1000	LP + 3000	SEM		Linear ^2^	Quadratic ^3^
Replicates ^4^		14	14	14	14				
Tibia weight (as is) (g)	4.07 ^b^	4.48 ^b^	5.60 ^a^	5.58 ^a^	5.94 ^a^	0.14	<0.001	<0.001	0.179
Tibia weight (dried) (g)	1.63 ^b^	1.80 ^b^	2.35 ^a^	2.28 ^a^	2.40 ^a^	0.06	<0.001	<0.001	0.045
Tibia dry matter (%)	39.79	40.5	42.3	40.9	40.5	0.41	0.406	0.545	0.117
Crude ash ^5^ (g/kg)	356 ^a^	294 ^b^	353 ^a^	378 ^a^	387 ^a^	5.21	<0.001	<0.001	<0.001
Phosphorus (g/kg)	34.3 ^bc^	25.1 ^d^	31.8 ^c^	39.9 ^ab^	41.0 ^a^	0.99	<0.001	<0.001	<0.001
Calcium (g/kg)	108 ^bc^	91.2 ^c^	114 ^ab^	123 ^ab^	133 ^a^	2.99	<0.001	<0.001	0.047

^1^ HPF: hypoallergenic phytate-free diet; LP: phytate-containing diets low in phosphorus; ^2^ Linear effects of supplementing increasing levels of phytase; ^3^ Quadratic effects of supplementing increasing levels of phytase; ^4^ Data are means of 14 cages with 2 birds per cage (pool); ^5^ Kruskal–Wallis test; ^a,b,c,d^ Within a row, means not sharing any common superscript are significantly different (*p* < 0.05).

**Table 5:** Effect of different inclusion levels of phytase on morphometry in jejunum and cecum of broiler chickens at 21 days of age.

							*p*-Value		
Treatment Groups ^1^	HPF	LP	LP + 500	LP + 1000	LP + 3000	SEM		Linear ^2^	Quadratic ^3^
Jejunum									
Replicates	9	10	10	9	11				
Villus length/BW ^4,5^ (μm/kg)	2594 ^a^	1667 ^b^	1393 ^b^	1551 ^b^	1395 ^b^	44.5	<0.001	0.102	0.201
Crypt depth/BW (μm/kg)	309 ^a^	253 ^b^	213 ^c^	203 ^c^	177 ^c^	5.75	<0.001	<0.001	<0.001
Villus/crypt ratio	8.36 ^a^	6.65 ^b^	6.57 ^b^	7.69 ^ab^	7.81 ^ab^	0.17	0.001	0.009	0.019
Cecum									
Replicates	12	12	11	12	12				
Crypt depth/BW (μm/kg) ^5^	537 ^a^	470 ^a^	384 ^ab^	394 ^ab^	321 ^b^	15.3	0.002	0.001	0.003

^1^ HPF: hypoallergenic phytate-free diet; LP: phytate-containing diets low in phosphorus; ^2^ Linear effects of supplementing increasing levels of phytase; ^3^ Quadratic effects of supplementing increasing levels of phytase; ^4^ BW: body weight; ^5^ Kruskal–Wallis test; ^a,b,c^ Within a row, means not sharing any common superscript are significantly different (*p* < 0.05).

## Data Availability

Data supporting the reported results is contained within the article.

## References

[1] Rodehutscord M., Rosenfelder P., Walk C.L., Kühn I., Stein H.H., Kidd M.T., Rodehutscord M. (2016). Update on phytate degradation pattern in the gastrointestinal tract of pigs and broiler chickens. Phytate Destruction. Consequences for Precision Animal Nutrition.

[2] Pallauf J., Rimbach G. (1997). Nutritional significance of phytic acid and phytase. Arch. Tierernahr..

[3] Angel C.R., Powers W.J., Applegate T.J., Tamim N.M., Christman M.C. (2005). Influence of Phytase on Water-Soluble Phosphorus in Poultry and Swine Manure. J. Environ. Qual..

[4] Cowieson A.J., Wilcock P., Bedford M.R. (2011). Super-dosing effects of phytase in poultry and other monogastrics. World’s Poult. Sci. J..

[5] Rodehutscord M., Walk C.L., Kühn I., Stein H.H., Kidd M.T., Rodehutscord M. (2016). Interactions between minerals and phytate degradation in poultry—Challenges for phosphorus digestibility assays. Phytate Destruction. Consequences for Precision Animal Nutrition.

[6] Dilger R.N., Adeola O. (2006). Estimation of true phosphorus digestibility and endogenous phosphorus loss in growing chicks fed conventional and low phytate soybean meals. Poult. Sci..

[7] Zeller E., Schollenberger M., Kühn I., Rodehutscord M. (2015). Hydrolysis of phytate and formation of inositol phosphate isomers without or with supplemented phytases in different segments of the digestive tract of broilers. J. Nutr. Sci..

[8] Rutherfurd S.M., Chung T.K., Moughan P.J. (2002). The effect of microbial phytase on ileal phosphorus and amino acid digestibility in the broiler chicken. Br. Poult. Sci..

[9] Cowieson A.J., Acamovic T., Bedford M.R. (2004). The effect of phytase and phytic acid on endogenous losses from broiler chickens. Br. Poult. Sci..

[10] Dersjant-Li Y., Awati A., Schulze H., Partridge G. (2015). Phytase in non-ruminant animal nutrition: A critical review on phytase activities in the gastrointestinal tract and influencing factors. J. Sci. Food Agric..

[11] Singh P.K. (2008). Significance of phytic acid and supplemental phytase in chicken nutrition: A review. World’s Poult. Sci. J..

[12] Kies A.K., Kemme P.A., Sebek L.B.J., van Diepen J.T.M., Jongbloed A.W. (2006). Effect of graded doses and a high dose of microbial phytase on the digestibility of various minerals in weaner pigs. J. Anim. Sci..

[13] Walk C.L., Bedford M.R., Santos T.T., Paiva D., Bradley J.R., Wladecki H., Honaker C., McElroy A.P. (2013). Extra-phosphoric effects of superdoses of a novel microbial phytase. Poult. Sci..

[14] Lee S.A., Nagalakshmi D., Raju M.V.L.N., Rama Rao S.V., Bedford M.R. (2017). Effect of phytase superdosing, myo-Inositol and available phosphorus concentrations on performance and bone mineralisation in broilers. Anim. Nutr..

[15] Lu H., Cowieson A.J., Wilson J.W., Ajuwon K.M., Adeola O. (2019). Extra-phosphoric effects of super dosing phytase on growth performance of pigs is not solely due to release of myo-inositol. J. Anim. Sci..

[16] Walters H.G., Coelho M., Coufal C.D., Lee J.T. (2019). Effects of increasing phytase inclusion levels on broiler performance, nutrient digestibility, and bone mineralization in low-phosphorus diets. J. Appl. Poult. Res..

[17] Haug W., Lantzsch H.J. (1983). Sensitive method for the rapid determination of phytate in cereals and cereal products. J. Sci. Food Agric..

[18] Short F., Gorton P., Wiseman J., Boorman K. (1996). Determination of titanium dioxide added as an inert marker in chicken digestibility studies. Anim. Feed Sci. Technol..

[19] Rutherfurd S.M., Chung T.K., Thomas D.V., Zou M.L., Moughan P.J. (2012). Effect of a novel phytase on growth performance, apparent metabolizable energy, and the availability of minerals and amino acids in a low-phosphorus corn-soybean meal diet for broilers. Poult. Sci..

[20] Walk C.L., Santos T.T., Bedford M.R. (2014). Influence of superdoses of a novel microbial phytase on growth performance, tibia ash, and gizzard phytate and inositol in young broilers. Poult. Sci..

[21] Beeson L.A., Walk C.L., Bedford M.R., Olukosi O.A. (2017). Hydrolysis of phytate to its lower esters can influence the growth performance and nutrient utilization of broilers with regular or super doses of phytase. Poult. Sci..

[22] Karami M., Karimi A., Sadeghi A.A., Zentek J., Goodarzi Boroojeni F. (2020). Effects of phytase and benzoic acid supplementation on growth performance, nutrient digestibility, tibia mineralization and serum traits in male broiler chickens. Livest. Sci..

[23] Sommerfeld V., Künzel S., Schollenberger M., Kühn I., Rodehutscord M. (2018). Influence of phytase or myo-inositol supplements on performance and phytate degradation products in the crop, ileum, and blood of broiler chickens. Poult. Sci..

[24] Smith K.A., Wyatt C.L., Lee J.T. (2019). Evaluation of increasing levels of phytase in diets containing variable levels of amino acids on male broiler performance and processing yields. J. Appl. Poult. Res..

[25] Walk C.L., Bedford M.R., Olukosi O.A. (2018). Effect of phytase on growth performance, phytate degradation and gene expression of myo-inositol transporters in the small intestine, liver and kidney of 21 day old broilers. Poult. Sci..

[26] Onyango E.M., Bedford M.R., Adeola O. (2005). Efficacy of an evolved Escherichia coli phytase in diets for broiler chicks. Poult. Sci..

[27] Selle P.H., Ravindran V., Partridge G.G. (2009). Beneficial effects of xylanase and/or phytase inclusions on ileal amino acid digestibility energy utilisation mineral retention and growth performance in wheat-based broiler diets. Anim. Feed Sci. Technol..

[28] Manobhavan M., Elangovan A.V., Sridhar M., Shet D., Ajith S., Pal D.T., Gowda N.K.S. (2016). Effect of super dosing of phytase on growth performance, ileal digestibility and bone characteristics in broilers fed corn-soya-based diets. J. Anim. Physiol. Anim. Nutr..

[29] Ravindran V., Morel P.C.H., Partridge G.G., Hruby M., Sands J.S. (2006). Influence of an E.coli-derived phytase on nutrient utilization in broiler starters fed diets containing varying concentrations of phytic acid. Poult. Sci..

[30] Li W., Angel R., Plumstead P.W., Enting H. (2021). Effects of limestone particle size, phytate, calcium source, and phytase on standardized ileal calcium and phosphorus digestibility in broilers. Poult. Sci..

[31] El-Hack M.E.A., Shafi M.E., Alghamdi W.Y., Abdelnour S.A., Shehata A.M., Noreldin A.E., Ashour E.A., Swelum A.A., Al-Sagan A.A., Alkhateeb M. (2020). Black soldier fly (*Hermetia illucens*) Meal as a promising feed ingredient for poultry: A comprehensive review. Agriculture.

[32] Selle P.H., Ravindran V.R., Caldwell A., Bryden W.L. (2000). Phytate and phytase: Consequences for protein utilisation. Nutr. Res. Rev..

[33] Cowieson A.J., Bedford M.R., Selle P.H., Ravindran V. (2009). Phytate and microbial phytase: Implications for endogenous nitrogen losses and nutrient availability. World’s Poult. Sci. J..

[34] Konietzny U., Greiner R., Caballero B., Trugo L., Finglas P. (2003). Phytic acid: Nutritional impact. Encyclopedia of Food Science and Nutrition.

[35] Cowieson A.J., Ruckebusch J.P., Sorbara J.O.B., Wilson J.W., Guggenbuhl P., Roos F.F. (2017). A systematic view on the effect of phytase on ileal amino acid digestibility in broilers. Anim. Feed Sci. Technol..

[36] Borda-Molina D., Zuber T., Siegert W., Camarinha-Silva A., Feuerstein D., Rodehutscord M. (2019). Effects of protease and phytase supplements on small intestinal microbiota and amino acid digestibility in broiler chickens. Poult. Sci..

[37] Moter V., Stein H.H. (2004). Effect of feed intake on endogenous losses and amino acid and energy digestibility by growing pigs. J. Anim. Sci..

[38] Rodehutscord M., Kapocius M., Timmler R., Dieckmann A. (2004). Linear regression approach to study amino acid digestibility in broiler chickens. Br. Poult. Sci..

[39] Ravindran V., Cabahug S., Ravindran G., Bryden W.L. (1999). Influence of microbial phytase on apparent ileal amino acid digestibility of feedstuffs for broilers. Poult. Sci..

[40] Veum T.L., Vitti D.M.S.S., Kebreab E. (2010). Phosphorus and calcium nutrition and metabolism. Phosphorus and Calcium Utilization and Requirements in Farm Animals.

[41] Ptak A., Józefiak D., Kierończyk B., Rawski M., Żyła K., Świątkiewicz S. (2013). Effect of different phytases on the performance, nutrient retention and tibia composition in broiler chickens. Arch. Anim. Breed..

[42] Paiva D., Walk C., McElroy A. (2014). Dietary calcium, phosphorus, and phytase effects on bird performance, intestinal morphology, mineral digestibility, and bone ash during a natural necrotic enteritis episode. Poult. Sci..

[43] Wu Y.B., Ravindran V., Thomas D.G., Birtles M.J., Hendriks W.H. (2004). Influence of phytase and xylanase, individually or in combination, on performance, apparent metabolizable energy, digestive tract measurements and gut morphology in broilers fed wheat-based diets containing adequate level of phosphorus. Br. Poult. Sci..

[44] Ziarat M.M., Kermanshahi H., Mogaddam H.N., Heravi R.M. (2020). Performance of an Escherichia coli phytase expressed in Lactococcus lactis on nutrient retention, bone traits and intestinal morphology in broiler chickens. J. Anim. Physiol. Anim. Nutr..

[45] Emami N.K., Naeini S.Z., Ruiz-Feria C.A. (2013). Growth performance, digestibility, immune response and intestinal morphology of male broilers fed phosphorus deficient diets supplemented with microbial phytase and organic acids. Livest. Sci..

[46] Smulikowska S., Czerwinski J., Mieczkowska A. (2010). Effect of an organic acid blend and phytase added to a rapeseed cake containing diet on performance, intestinal morphology, caecal microfloral activity and thyroid status of broiler chickens. J. Anim. Physiol. Anim. Nutr..

[47] Lillehoj H.S. (1993). Avian gut-associated immune system: Implication in coccidial vaccine development. Poult. Sci..

[48] Bedford M.R., Walk C.L., Walk C.L., Kühn I., Stein H.H., Kidd M.T., Rodehutscord M. (2016). Reduction of phytate to tetrakisphosphate (IP_4_) to trisphosphate (IP_3_), or perhaps even lower, does not remove its antinutritive properties. Phytate Destruction. Consequences for Precision Animal Nutrition.

